# Supplementary material for: Identifying wastewater management tradeoffs: Costs, nearshore water quality, and implications for marine coastal ecosystems in Kona, Hawai‘i
Source: PLoS One. 2021 Sep 8;16(9):e0257125. doi: 10.1371/journal.pone.0257125 (PMC8425575; doi:10.1371/journal.pone.0257125)
Supplement: S1 Fig — Risk scores were calculated by [25]. (DOCX) [file pone.0257125.s001.docx]

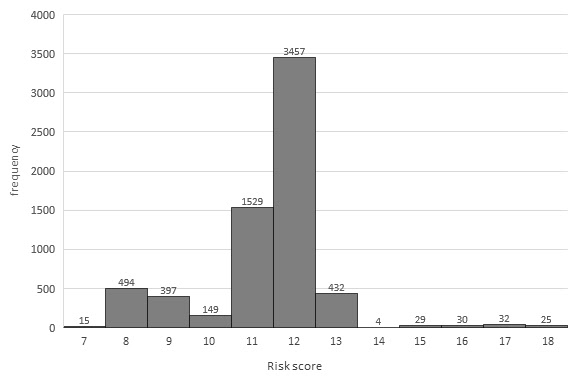


**S1 Fig. Histogram of OSDS risk scores across the Keauhou basal aquifer. Risk scores were calculated by [25].**
