# Supplementary material for: Identifying wastewater management tradeoffs: Costs, nearshore water quality, and implications for marine coastal ecosystems in Kona, Hawai‘i
Source: PLoS One. 2021 Sep 8;16(9):e0257125. doi: 10.1371/journal.pone.0257125 (PMC8425575; doi:10.1371/journal.pone.0257125)
Supplement: S2 Fig — The vertical cross section A-A’ consists of 21 layers. The top layer follows the local topography and bathymetry and the bottom layer follows a flat elevation of 550 m below mean sea level. The bottom elevation of the top layer is set to 1 m below mean sea level to ensure dry cells were not produced. The layer thickness gradually increases, where the uppermost layers are thinnest. (DOCX) [file pone.0257125.s002.docx]

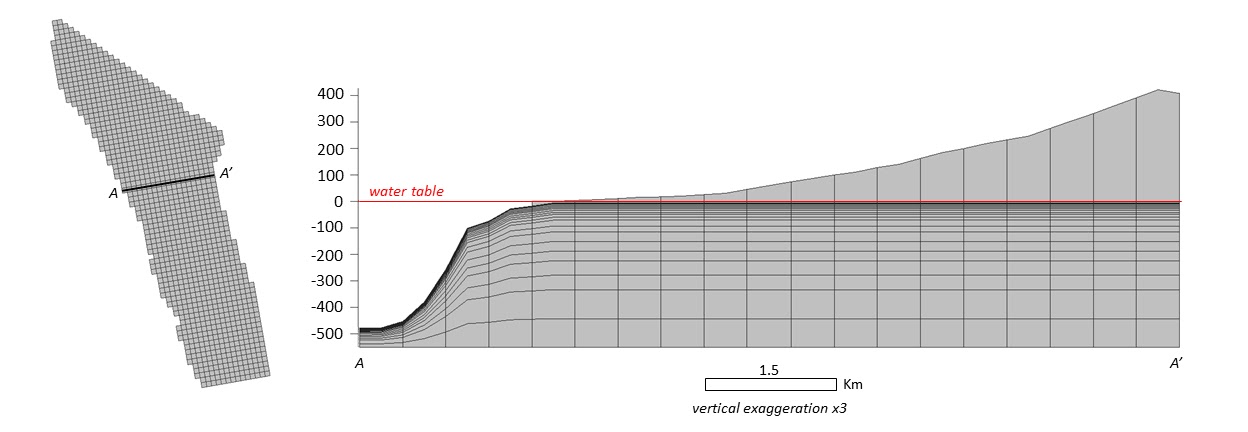


**S2 Fig. Numerical model grid setup for the Keauhou basal aquifer. The vertical cross section A-A’ consists of 21 layers. The top layer follows the local topography and bathymetry and the bottom layer follows a flat elevation of 550 m below mean sea level. The bottom elevation of the top layer is set to 1 m below mean sea level to ensure dry cells were not produced. The layer thickness gradually increases, where the uppermost layers are thinnest.**
