# Supplementary material for: Identifying wastewater management tradeoffs: Costs, nearshore water quality, and implications for marine coastal ecosystems in Kona, Hawai‘i
Source: PLoS One. 2021 Sep 8;16(9):e0257125. doi: 10.1371/journal.pone.0257125 (PMC8425575; doi:10.1371/journal.pone.0257125)
Supplement: S1 Table — (DOCX) [file pone.0257125.s003.docx]

**S1 Table. Number of cesspools converted by type of upgrade for each management scenario**

|  | **NUMBER OF CESSPOOLS CONVERTED** | | | | | | | | | |  |
| --- | --- | --- | --- | --- | --- | --- | --- | --- | --- | --- | --- |
| **Scenario** | **Septic** | | | | | **ATU** | | | | | **Total** |
|  | *1 BR* | *2 BR* | *3 BR* | *4 BR* | *5 BR* | *1 BR* | *2 BR* | *3 BR* | *4 BR* | *5 BR* |  |
| Present | 0 | 0 | 0 | 0 | 0 | 0 | 0 | 0 | 0 | 0 | 0 |
| Future permitted | 0 | 0 | 0 | 0 | 0 | 0 | 0 | 0 | 0 | 0 | 0 |
| All ATU upgrade | 0 | 0 | 0 | 0 | 0 | 233 | 1334 | 4088 | 973 | 625 | 7253 |
| WWTP upgrade | 0 | 0 | 0 | 0 | 0 | 0 | 0 | 0 | 0 | 0 | 0 |
| Target low efficiency | 180 | 965 | 2991 | 682 | 472 | 53 | 369 | 1097 | 291 | 153 | 7253 |
| Target high efficiency | 180 | 965 | 2991 | 682 | 472 | 53 | 369 | 1097 | 291 | 153 | 7253 |
| Target low efficiency + WWTP upgrade | 180 | 965 | 2991 | 682 | 472 | 53 | 369 | 1097 | 291 | 153 | 7253 |
| Target high efficiency + WWTP upgrade | 180 | 965 | 2991 | 682 | 472 | 53 | 369 | 1097 | 291 | 153 | 7253 |
