# Supplementary material for: Identifying wastewater management tradeoffs: Costs, nearshore water quality, and implications for marine coastal ecosystems in Kona, Hawai‘i
Source: PLoS One. 2021 Sep 8;16(9):e0257125. doi: 10.1371/journal.pone.0257125 (PMC8425575; doi:10.1371/journal.pone.0257125)
Supplement: Attachment — Submitted filename: Review.docx [file pone.0257125.s004.docx]

In this manuscript, the authors aim to assess wastewater management scenarios and trade-offs for improving water quality on coral reefs in Kona, Hawaii. The question is relevant and interesting, and the dataset and modelling approach are impressive. In general, I find the text to be well-writte, albeit could improve in conciseness/clarity, and commend the authors for the work that obviously went into this manuscript. My biggest concern involves the trade-off analysis, along with some of the terminology to be misleading, and some more minor clarification needed. Please find my major and minor comments below.

Major comments:

Most of my major concerns involve the trade-off analysis. I find the spider diagram/ranking approach to be a bit convoluted. It seems that all data is available to do (e.g., Table 4) a proper cost-benefit analysis and it is unclear to me why this was not done? While the spider diagrams are potentially useful as a supplemental figure, determining the benefit (e.g., improvement in water quality) per unit cost of each wastewater management scenario seems a more straightforward and justifiable approach. This would provide a clearer, more easily explainable prioritisation of which scenario provides the greatest benefit per dollar, allowing for more concrete recommendations. Beher et al. 2016 Prioritising catchment management projects to improve marine water quality exemplifies the sort of approach I am referring to in a similar, water quality context.

I find the framing of “coral reef habitat impact” to be misleading – impact, to me, implies a change in condition. As the authors do not measure or model any change in coral reef condition due to nutrient exposure (which is difficult to do!) I would suggest removing reference to impact and framing around nutrient exposure or potential risk of impact on coral reefs. Some discussion around the fact that we really don’t know much about how corals would respond would be useful in the limitations section of the discussion.

Minor comments:

General minor comments:

1. Ensure that acronyms are all defined when they are presented
2. I think in many cases the writing could be a bit more concise – a read for conciseness
3. Check citation formatting as some intext citations were not clear – but best to check journal guidelines (maybe it’s correct?).

Line 58: the phrasing “most important” is subjective – I would consider rewording

Line 93: Can you give an example of a ground-water dependent ecosystem? I am not as familiar so this would help clarify what the authors are talking about

Line 133: Very minor, but wondering why it is referred to as “mountain-to-sea” as opposed to the more common “ridge-to-reef” framing.

Line 154: Capitalise “Scenarios”

Line 164: I find the “risk scores” aspect described in the methods to be very unclear currently. How were they determined? Is the 12 threshold (line 215) basically arbitrary? What are the implications of some of the decisions made around the risk scores and how might they change results?

Table 1. I wish there was more detail about the scenarios here so you could understand them from just the table quickly instead of trying to dig through the methods. For example – for the All ATU upgrade, I thought it would be All-ATU under cesspool conversion and a N under WWTP upgrade – or is the “upgrade”referring to WWTP? Maybe the name is misleading? The difference between Scenario 1 and 2 is also confusing until you read through the text. In general, the table brought up a lot of confusion for me until I read through the text, but even then some aspects weren’t particularly clear. A quick description column would help.

Line 248: I assume the 21 layers are spatial layers? Is there a supplemental table with a description of these?

Line 286: Why was 1.2 km from the shoreline chosen in the diffusion model? A brief justification would help.

Line 327: was confused what BR stood for – it is introduced later – make sure introduced where first mentioned.

Line 502: Beyond the scope of this paper – but just a note that it seems this analysis could be upgraded fairly easily to a spatial prioritisation. This would be very interesting extension of the work that I hope the authors look into!

Line 549: “A shift towards turf-algal dominated systems is seen in the scenarios where nutrient inputs are increased” – this seems like it came out of no where? I don’t see any mention of measuring changes in benthic cover from nutrients, from what I can tell it is only changes in nutrient concentrations so then I am confused on how shifts to turf-dominated systems are detected?

Figures – In general the figures are very nicely done and laid out. For the figures describing nutrient decreases/increases – why are they binned into high/medium/low? What values are considered in each of these bins/how were they determined? Why not sure a continuous scale or quartiles with actual values?
